# Factors associated with police shooting mortality: A focus on race and a plea for more comprehensive data

**DOI:** 10.1371/journal.pone.0259024

**Published:** 2021-11-10

**Authors:** Justin Nix, John A. Shjarback

**Affiliations:** 1 School of Criminology and Criminal Justice, University of Nebraska Omaha, Omaha, NE, United States of America; 2 Department of Law and Justice Studies, Rowan University, Glassboro, NJ, United States of America; London School of Economics, UNITED KINGDOM

## Abstract

**Objectives:**

To quantify nonfatal injurious police shootings of people and examine the factors associated with victim mortality.

**Methods:**

We gathered victim-level data on fatal and nonfatal injurious police shootings from four states that have such information publicly available: Florida (2009–14), Colorado (2010–19), Texas (2015–19), and California (2016–19). For each state, we examined bivariate associations between mortality and race/ethnicity, gender, age, weapon, and access to trauma care. We also estimated logistic regression models predicting victim mortality in each state.

**Results:**

Forty-five percent of these police shooting victims (N = 1,322) did not die. Black–white disparities were more pronounced in nonfatal injurious police shootings than in fatal police shootings. Overall, Black victims were less likely than white victims to die from their wound(s). Younger victims were less likely to die from their wound(s), as well as those who were unarmed.

**Conclusions:**

Racial and age disparities in police shootings are likely more pronounced than previous estimates suggest.

**Policy implications:**

Other states should strongly consider compiling data like that which is currently being gathered in California. Absent data on nonfatal injurious police shootings–which account for a large share of deadly force incidents–researchers and analysts must be cautious about comparing and/or ranking jurisdictions in terms of their police-involved fatality rates.

## Introduction

Thanks to recent advances in data tracking police-caused fatalities, we know that roughly 1,000 people are killed by police gunfire each year in the United States [1]. We also know that Black Americans are disproportionally killed, as they make up 13% of the US population yet 25% of those killed by police. For example, Edwards and colleagues drew on *Fatal Encounters* data to show that “the risk of being killed by police, relative to white men, is between 3.2 and 3.5 times higher for Black men and between 1.4 and 1.7 times higher for Latino men” [2]. In a separate study analyzing data from *Mapping Police Violence*, Schwartz and Jahn found that “across all [382 metropolitan statistical areas], Black people were 3.23 times more likely to be killed compared to white people…Latinx people were 1.05 times more likely, though this [disparity] was not statistically significant” [3]. However, a key limitation of both studies (and others using these or similar datasets like *The Washington Post’s* “Fatal Force”) is that they omit a significant number of incidents involving the use of deadly force by police officers.

The late James Fyfe defined police use of deadly force as “physical force capable of or likely to kill,” noting that “it does not always kill” and “the true frequency of police decisions to employ firearms as a means of deadly force…can best be determined by considering woundings and off-target shots as only fortuitous variations of fatal shootings” [4]. In other words, every time an officer discharges their firearm at someone, the officer is using deadly force. Studies from the 1970s through present day indicate that those killed by police gunfire are but a portion of the total number of people shot or shot at. For example, 44% of those shot by officers from the New York Police Department died from 1971–1975 [5], 33% of those shot by officers from St. Louis Metropolitan Police Department died from 2003–2012 [6], and 49% of individuals struck by police gunfire throughout the state of Texas died from 2016–2017 [7]. Across 47 large U.S. jurisdictions from 2010 to 2016, only 31% of police shootings resulted in a fatality, and there was a great deal of jurisdictional variation in terms of fatality rate [8].

Given that police gunfire is responsible for more than 94% of police-caused fatalities nationwide [3], it is likely that recent scholarship drawing on datasets like *Fatal Encounters*, *Mapping Police Violence*, or *The Washington Post’s* “Fatal Force” severely underestimates the rate at which police use deadly force. Indeed, far less is known about police shootings that do not result in death due to the absence of comprehensive data. For example, on August 23, 2020, a 29-year-old Black man named Jacob Blake was shot several times by a Kenosha, Wisconsin police officer [9]. Though Blake did not die, he was nonetheless the victim of deadly force by police. At present, however, neither Blake’s name nor details of the circumstances surrounding this shooting will appear in any dataset that only catalogues *deaths* at the hands of American police.

The aforementioned databases, while an improvement over the federal government’s systems that underestimate fatalities by roughly 50% [10–13], still represent an incomplete and likely non-random portion of all occasions where deadly force is employed by police officers. While there is some degree of chance between whether a person who is shot lives or dies [14], it is possible that a variety of situational, organizational, and/or ecological characteristics influence the likelihood of a police shooting being fatal, and, therefore, appearing in publicly available datasets. Any analysis of police deadly force restricted to data that only include fatalities may produce results that are statistically biased by factors that influence mortality, such as whether bullets strike vital organs [12], whether police officers administer first aid or engage in “scoop and run” practices [15, 16], and whether an adult trauma care center is nearby [17, 18]. Regarding race and the ecology of place, and given disparities in access to quality hospitals across geographic areas (e.g., urban versus suburban versus rural) [19], among other factors, mortality rates in police shootings may meaningfully differ among racial/ethnic groups [7]. Indeed, one analysis of fatal and nonfatal police shootings in nine U.S. jurisdictions revealed that Black victims were more likely to survive [20]. Thus, it is possible that extant research may have underestimated racial disparities in police use of deadly force.

The current study leverages data on fatal and injurious police shootings from four states–Florida, Texas, Colorado, and California–to examine the racial/ethnic composition of mortality in police shootings, while accounting for several demographic, situational, and ecological characteristics (e.g., county-level access to Level I or II adult trauma centers). Findings advance the state of knowledge on police shootings and reveal a critical need for better data on police uses of deadly force that do not result in death.

## Methods and data

Data from four states allow for an evaluation of both fatal and injurious police shootings of people: Florida, Colorado, Texas, and California. Collectively, these 4 states have accounted for 2,150 of 6,329 (34%) fatal police shootings since 2015 (per *Washington Post* “Fatal Force” data, accessed 6/8/2021). In terms of the raw number of people fatally shot by police, California ranks 1^st^ (939), Texas 2^nd^ (565), Florida 3^rd^ (420), and Colorado 5^th^ (226). In per capita terms, Colorado ranks 5^th^ (6.2 per million), California ranks 18^th^ (3.7 per million), Florida ranks 26^th^ (3.1 per million), and Texas ranks 28^th^ (3.0 per million).

Florida’s data comes from the *Tampa Bay Times*’ “Why Cops Shoot” database. Drawing on freedom of information requests, media reports, and court documents, this journalist-compiled data records 823 people shot by police from January 1, 2009 through December 31, 2014 (more details about the data sources are provided in the supplemental materials). The three remaining data sources are derived from state government systems. Colorado’s Division of Criminal Justice maintains a database of 404 people shot and injured or killed between January 1, 2010 and June 30, 2019. Texas’ database, which is maintained by the Office of the Attorney General, catalogues 752 people shot and injured or killed by police between September 1, 2015 through December 31, 2019. Finally, California Department of Justice’s “URSUS” use of force reporting system documents 989 people who were shot and injured or killed by police between January 1, 2016 and December 31, 2019.

As a simple data reliability check, we compared the number of fatal shootings in our Colorado, Texas, and California data from 2016 to 2019 to the number captured in each state respectively during the same period by *The Washington Post’s* “Fatal Force” database (WAPO; accessed 9/13/2021). Our Colorado data indicate 141 fatal shootings occurred during this period; WAPO indicates 142. Our Texas data show 371 fatal shootings during this period; WAPO shows 343. Finally, our California data indicate 549 fatal shootings during this period; WAPO indicates 548. These results should minimize concern about underreporting by agencies.

### Measures

**Fig 1:**
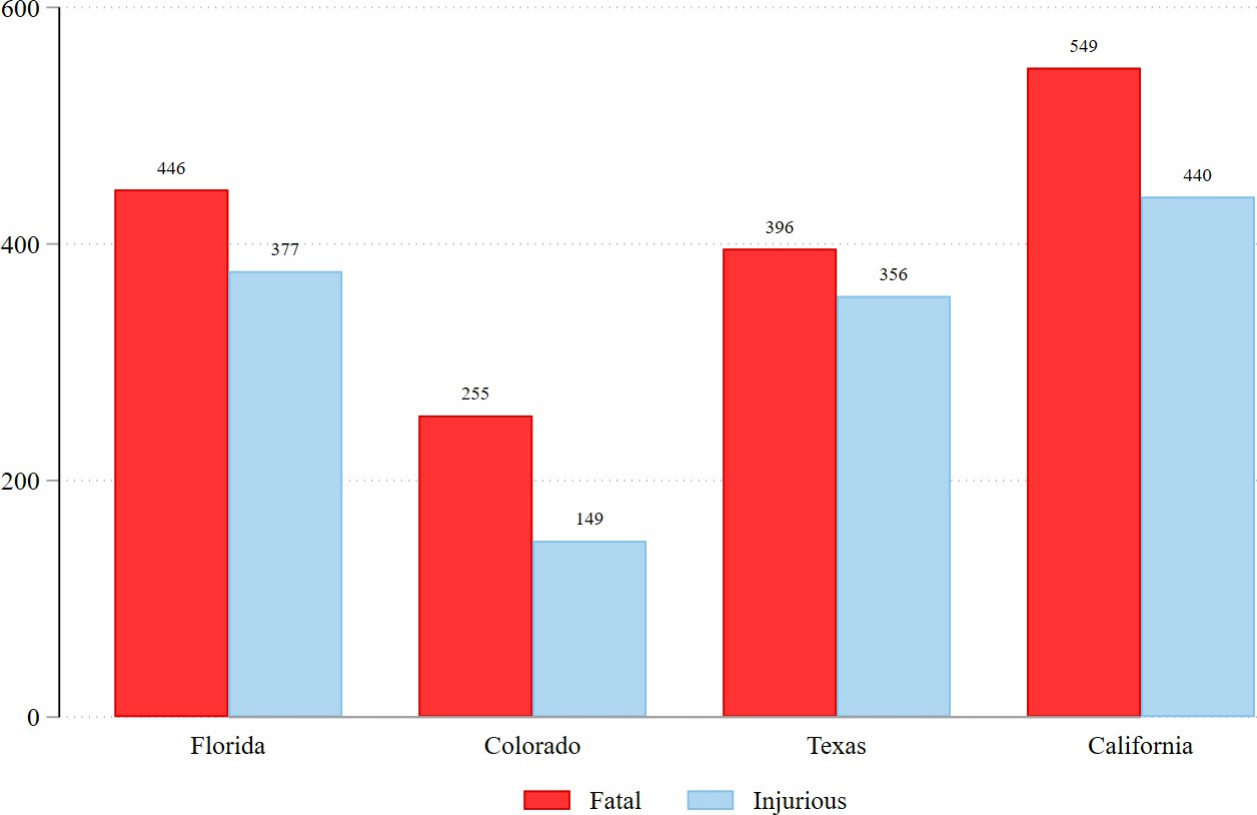
Fatal and injurious police shootings in each state.

The dependent variable is *mortality* and it was measured dichotomously (1 = fatal; 0 = injurious). As shown in Fig 1, a large share of police shootings in each state resulted in nonfatal injuries. Across the four states, 44.5% of those shot by police were injured (n = 1,322) while 55.5% were killed (n = 1,646).

Several victim demographics, situational, and ecological covariates were collected that allow for uniform measurement across states. Victim *race/ethnicity* was categorized into white (reference group), Black, Hispanic, and other; we relied strictly on the classification provided by each dataset. Victim *gender* was broken down into male or female. Victim *age* includes the following categories: 25 and under (reference group), 26–35, 36–45, and 46 and older. Note that California provided pre-binned age categories; Florida, Colorado, and Texas provided the precise age of each victim, which permitted use of a continuous *age* variable in state-specific analyses (see S3–S5 Tables in S1 File). All states provided information on whether the victim was *armed* with a potentially deadly weapon (e.g., firearm, blunt object, cutting instrument) (1 = yes) or not.

All four states provide geographic information sufficient to identify the county in which each shooting occurred. The Trauma Center Association of America [21] was then used to determine whether the county in which the shooting occurred possessed either a *Level I or II Adult Trauma Center*, which was measured dichotomously (1 = yes; 0 = no; results were substantively the same when we used an ordinal measure–see *S2 Table* in S1 File). Three states–Florida, Colorado, and California–indicate whether the victim displayed signs of *mental illness* (1 = yes). Additionally, a dichotomized measure of each county’s urbanicity was collected from the US Department of Agriculture’s Economic Research Service (ERS) [22]. The ERS’s 2013 Rural-Urban Continuum Code classifies counties into metropolitan (1 = yes) versus non-metropolitan based on population size and geographic proximity to metropolitan areas. Lastly, California’s URSUS system includes a field that indicates the location of the gunshot wound. *Head/Torso shot* measured whether police gunfire struck the victim’s head, chest, or upper back (1 = yes), where the odds of striking a vital organ are greater, thus increasing the likelihood of mortality [12].

### Analytic strategy

Our analysis proceeded in several steps. First, we present descriptive statistics and a comparative analysis of racial/ethnic disparities in fatal and injurious police shootings of people. We then cross tabulated shooting outcomes (fatality/injury) with victim race/ethnicity, gender, age, deadly weapon, county access to trauma care, urbanicity, mental illness (Florida, Colorado, and California only), and location of gunshot wound on the victim’s body (California only; see S1 Table in S1 File). County-level mortality rates were plotted to visualize variation in survivability across geographic space. Next, we estimated logistic regression models predicting police shooting fatalities using a pooled sample of cases from all four states while controlling for victim, situational, and ecological characteristics. Listwise deletion of 76 cases with missing information on some of the covariates reduced the sample size to N = 2968 for the multivariable analysis. The mean variance inflation factor for the main model was 1.15 and the condition number was 20.38, indicating multicollinearity was not a concern [23]. In addition to the pooled sample analysis, we estimated a series of state-specific logistic regression models predicting police shooting fatalities in S3–S6 Tables in S1 File, which in some instances included additional covariates that were not available in every state (e.g., Texas’ data did not indicate whether victims displayed signs of mental illness, thus prohibiting us from including such a covariate in our analysis of the pooled sample).

## Results

All told, 1,322 individuals were shot *but not killed* by police (44.5%). Colorado’s police shooting victims had the highest mortality rate at 63%, followed by California (56%), Florida (54%), and Texas (53%; see Fig 1). Mortality rates differed greatly across counties within states (see *S1–S4 Figs* in S1 File). Metropolitan counties where large, highly populated cities are located tended to have lower mortality rates (i.e., increased likelihood of individuals surviving their gunshot wounds from police).

To assess racial/ethnic disparities in fatal and injurious police shootings, we calculated a series of odds ratios using the racial/ethnic composition of the general population using estimates from the US Census Bureau’s American Community Survey (ACS) as benchmarks. While other benchmarks may seem better suited for “making sense” of any observed disparities, particularly those that capture transactional behavior (e.g., level/threat to officers) on the part of citizens, these measures were not consistently available across states. Furthermore, these other benchmarks–such as police-citizen interactions or arrests–are contaminated by police discretion (i.e., to interact with people). Thus, conditioning on them would (1) block a mediating path between victim race and the outcome being examined here (police shooting) and (2) induce collider bias [24]. Five-year ACS estimates from 2010–2014 were used for Florida, while 2015–2019 estimates were used for Texas, Colorado, and California–largely due to ease and availability. Odds ratios were calculated using the following formula:

$$\frac{\left[[Black|Hispanic]\;share\;of\;police\;shootings÷\left[Black|Hispanic\right]\;share\;of\;population\right]}{\left[White\;share\;of\;police\;shootings\;÷White\;share\;of\;population\right]}$$

Odds ratios greater than 1.0 indicate that Black (or Hispanic) people were *more likely* than white people to be shot by police. Odds ratios less than 1.0 indicate that Black (or Hispanic) people were *less likely* than white people to be shot by police. To be clear, these odds ratios are merely descriptive statistics indicating the probability of a randomly sampled Black or Hispanic person being shot by a police officer relative to a randomly sampled white person. They should not be interpreted as evidence that police officers react differently to people of one group versus another during encounters. Such a determination would require complete information on all police-citizen interactions in each state, including those that did not result in police shootings [20, 24].

**Fig 2:**
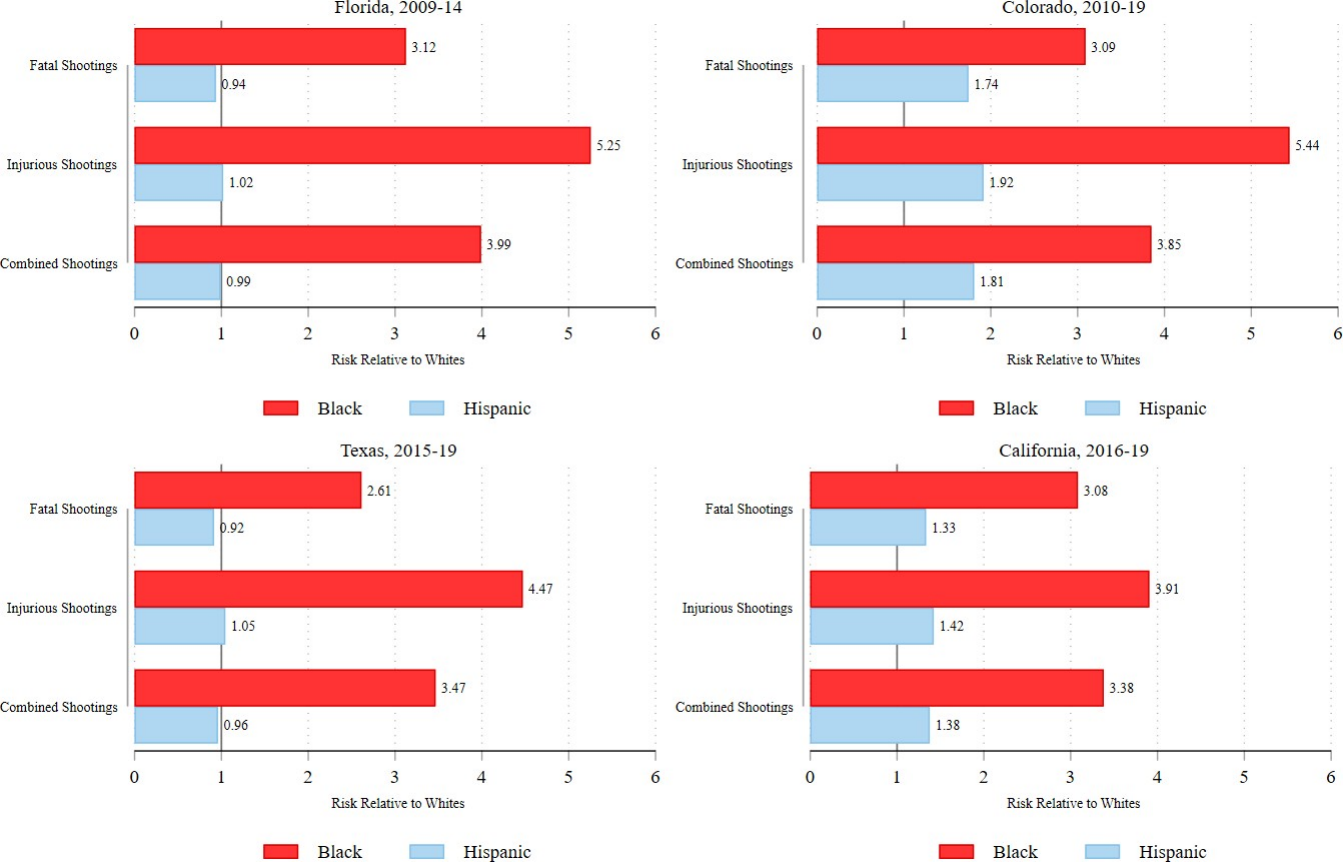
Racial disparities in fatal and injurious police shootings in each state. * Population estimates for FL are 2010–14 ACS 5-year estimates. Population estimates for CO, TX, and CA are 2015–19 ACS 5-year estimates.

Fig 2 displays the Black-white and Hispanic-white disparities in police shootings for each state, respectively. In every state, racial disparities are more pronounced in injurious police shootings than fatal police shootings. For example, in Florida from 2009 to 2014, Black people were approximately 3.12 times as likely as white people to be fatally shot by police, yet 5.25 times as likely as white people to be nonfatally shot. In California from 2016 to 2019, Black people were 3.08 times as likely as white people to be fatally shot by police, and 3.91 times as likely to be nonfatally shot. Shifting to Hispanic-white disparities, Fig 2 shows they were quite small in Florida and Texas. In Colorado and California, Hispanic people were more likely than whites to be shot by police, but the disparities were less extreme than Black-white disparities in those states. And here again, in these states, Hispanic-white disparities in injurious police shootings were larger than in fatal police shootings. The main takeaway from Fig 2 is that in all four states, we would *underestimate* Black-white disparities in police use of deadly force if we only had access to fatal police shooting data.

**Fig 3:**
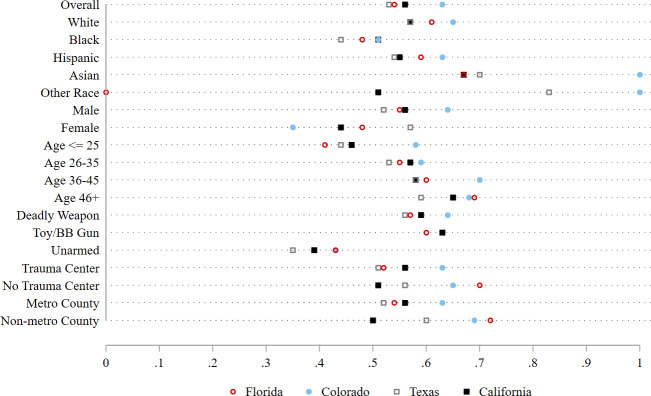
Proportion of shootings that were fatal across each covariate, in each state.

Before turning the results of the logistic regression models predicting shooting mortality in the pooled sample, it is informative to look at the proportion of shootings that were fatal across each covariate in each state. See Fig 3 (raw *N*s are provided in S1 Table in S1 File). Note that these are merely raw comparisons and differences may or may not be statistically meaningful (we withhold tests of statistical significance until the logistic regression models presented in Table 1). In each state, Black victims appear less likely than white victims to die from a gunshot wound(s) inflicted by police. Males appear more likely than females to die from their wounds in Florida, Colorado, and California, but not in Texas. Victims 25 or younger appear more likely to survive police shootings than older victims in each state. Also, in each state, unarmed victims appear more likely to survive police shootings than those who were armed with a potentially deadly weapon. Victims who were shot in counties that did not have a Level I or II Adult trauma center appear more likely to die than those shot in counties with them in Florida, Colorado, and Texas. In California, however, the opposite was true. Similarly, victims who were shot in metropolitan counties appear less likely to die in Florida, Colorado, and Texas, but slightly more likely to die in California.

**Table 1:** Logistic regression models predicting the mortality of police shootings in the pooled sample.

	Model 1			Model 2		
	*b*	SE	dy/dx (95% CI)	*b*	SE	dy/dx (95% CI)
Black victim ^a^	-.490***	.099	-.121 (-.169, -.074)	-.277*	.108	-.069 (-.121, -.016)
Hispanic victim ^a^	-.133	.086	-.032 (-.074, .009)	.040	.106	.010 (-.041, .060)
Other victim ^a^	.189	.235	.044 (-.062, .151)	.282	.241	.067 (-.042, .177)
Male victim	—	—	—	.080	.158	.020 (-.057, .097)
Age 26–35 ^b^	—	—	—	.343***	.087	.085 (.043, .128)
Age 36–45 ^b^	—	—	—	.531***	.109	.131 (.079, .183)
Age 46+ ^b^	—	—	—	.665***	.149	.163 (.094, .232)
Weapon	—	—	—	.649***	.105	.161 (.110, .211)
Trauma care	—	—	—	-.018	.110	-.005 (-.058, .049)
Metro county	—	—	—	-.160	.193	-.039 (-.130, .052)
Colorado ^c^	—	—	—	.126	.179	.030 (-.054, .115)
Texas ^c^	—	—	—	-.199	.118	-.049 (-.106, .008)
California ^c^	—	—	—	-.095	.105	-.023 (-.074, .027)
Intercept	.396***	.062	—	-.454	.237	—
N	2,940			2,892		
Wald χ^2^	32.41***			117.25***		

Abbreviations: SE = Robust Standard Errors clustered on 246 counties; dy/dx = Average marginal effects showing the discrete change in the outcome (fatality) when moving from the reference category (estimated using *margins* command in Stata v15).

Reference categories are

^a^ White victim,

^b^ Age 25 and under, and

^c^ Florida, respectively.

* *p* < .05,

** *p* < .01,

*** *p* < .001.

Table 1 presents the results from logistic regression models predicting police shooting fatalities in all four states (i.e., the pooled sample). A series of dummy variables were created to measure race/ethnicity: *Black*, *Hispanic*, and *other* (combining the “Other” and “Asian” categories in Fig 3); *white* serves as the reference group for all analyses. Additionally, *deadly weapon* and *toy/BB gun* were combined to create a dummy variable indicating a real or perceived *weapon* (1 = yes, 0 = no), given the realistic nature of most of the toy/replica guns [25]. In Model 1, we included only the race/ethnicity covariates and the intercept term. Here, we observe that Black people are significantly less likely than white people to die when they are shot by police officers (*b* = -.490, *p* < .001). Hispanic victims and those from other racial/ethnic groups did not differ significantly from white victims in terms of police shooting mortality. The *dy/dx* column shows the discrete change in the outcome (i.e., fatality) when moving from the reference category and holding other covariates at their means (95% confidence intervals are reported in parentheses). This model estimates that in this sample, the average Black person was about 12 percentage points less likely to die when shot by a police officer than the average white person (95% CI: -16.9, -7.4).

**Fig 4:**
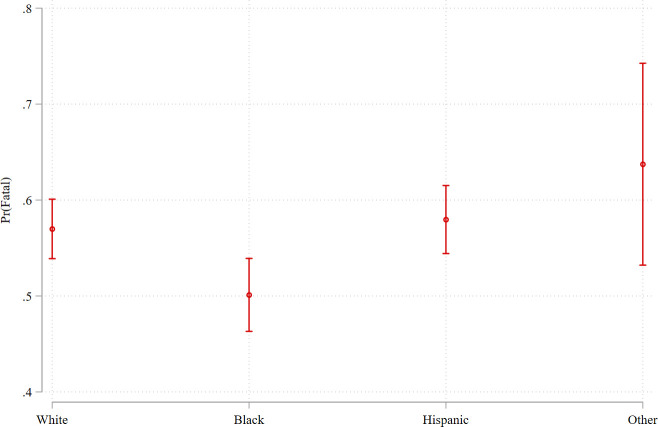
Adjusted predictions of Pr(*fatal*) by race/ethnicity in the pooled sample (with 95% confidence intervals).

In Model 2, we included covariates for gender, age, deadly weapon, county-level access to Level I/II trauma centers, county urbanicity, and state. The magnitude of the *Black victim* coefficient was reduced, but remained statistically significant (*b* = -.277, *p* < .05). This model estimates that the average Black person was about 7 percentage points less likely to die when shot by a police officer than the average white person (95% CI: -12.1, -1.6). Fig 4 plots the marginal effects of race/ethnicity on the probability of a police shooting being fatal. On average, white victims of police shootings die 57% of the time (95% CI: 53.9%, 60.1%), whereas black victims die 50.1% of the time (95% CI: 46.3%, 53.9%). Meanwhile, Hispanic victims of police shootings die 58.0% of the time (95% CI: 54.4%, 61.5%), and victims of other racial/ethnic groups die 63.7% of the time (95% CI: 53.2%, 74.3%).

We also observe in Model 2 of Table 1 that older victims are generally less likely to survive police shootings than younger victims. Recall that *25 or younger* serves as the reference group. Those who were between the ages of 26 and 35 were 8.5 percentage points more likely to die when shot by police (95% CI: 4.3, 12.8). Those who were between the ages of 36 and 45 were 13.1 percentage points more likely to die (95% CI: 7.9, 18.3), and those who were 46 or older were 16.3 percentage points more likely to die (95% CI: 9.4, 23.2). Each of these differences was statistically significant at the *p* < .001 level. Lastly, the model indicates that victims who were in possession of a potentially deadly weapon (e.g., firearm, toy/replica firearm, blunt object, cutting instrument) were 16 percentage points more likely to die than those who were unarmed (95% CI: 11.0, 21.1). This difference was statistically significant at the *p* < .001 level. Although the coefficients for access to trauma care (*b* = -.018) and urbanicity (*b* = -.160) were in the expected direction–that is, shooting victims were less likely to die in counties with at least one Level I or II adult trauma center and those counties considered metropolitan–they were not statistically significant at the .05 alpha level.

### Public health implications

Compared to the attention paid to fatal police shootings in recent years [2, 3, 26, 27], nonfatal injurious police shootings are far less scrutinized. Given that there are likely several hundred–if not more than one thousand–people shot *but not killed* by police each year in the United States, this represents a serious public health oversight. There are well-documented “spillover” effects in the aftermath of police killings: nearby students perform more poorly in the classroom and report higher levels of emotional disturbance [28], nearby residents exhibit greater risk of high blood pressure [29], and in some cases, nearby Black residents exhibit higher levels of depression and post-traumatic stress disorder [30]. At present, however, we are unable to assess the cumulative societal cost of police shootings, as we lack data on injurious shootings. These include, at a minimum, lost wages, medical bills, and debilitating and chronic health problems, as in the case of Jacob Blake who is now paralyzed [31]. American taxpayers are also impacted, as they ultimately pay the lion’s share of medical costs stemming from gun violence [32]. Public records also show thirty-one large US cities paid out more than three billion dollars over the past ten years to settle misconduct lawsuits–often brought on by police shootings and other deaths in custody or use of force incidents [33, 34]. From a public policy perspective, if we want to minimize police use of deadly force, we need comprehensive measures–not just data on those occasions where victims succumb to their gunshot wounds. Such data will permit future research to better examine the situational [20], organizational (e.g., stricter departmental policies regarding the sanctity of human life and using firearms as an absolute last resort) [16, 35], and ecological factors associated not only with police use of deadly force but also mortality [36].

One of the most basic questions is whether, as a group, the victims of nonfatal injurious police shootings resemble victims of fatal police shootings in terms of race/ethnicity, gender, age, and so on. To answer this question, our study leveraged data from four states for which several years of data on fatal *and* nonfatal injurious police shootings are publicly available. We found that in the four states examined, Black victims appear more likely to survive police shootings than white victims. If this pattern holds true nationwide, it means we have *underestimated* the extent of racial disparities in police use of deadly force based on the racial makeup of the population [2, 3, 26]. However, it is unclear whether regional patterns are consistent across the country; in the south, for example, there tend to be more Black Americans residing in rural areas. Older victims were more likely to die when shot by the police than those 25 or younger in all four states–a pattern that mirrors mortality in non-police shootings [37]. Prior research suggests the risk of being killed by police is highest for all racial groups between the ages of 20 and 35 [26]. Our findings suggest age disparities in who is shot by police may be even more pronounced than previously thought, as *FatalEncounters*.*org* and similar databases that track police-involved fatalities are likely capturing a larger percentage of shootings involving older victims and a relatively smaller percentage of shootings involving younger victims. The same could even be true of the most controversial police shootings: those involving unarmed persons or “threat perception failures” in which officers mistake an item (e.g., cell phone) for a gun [38].

### Limitations

This study is not without limitations. First, our analysis was restricted to four states for which fatal *and* injurious police shooting data are publicly available. Though it is notable that our sample included the three most populous states in the US, we cannot be sure these results generalize to other states. Second, with the exception of the Florida police shooting data, our study was largely reliant on official accounts of police shooting incidents, in which there is an opportunity for officers and departments to classify critical information about events (e.g., levels of threat and resistance) as justifiable and in accordance with administrative policy [39]. Additionally, officers’ memories of a shooting incident may be filtered through perceptual distortions and may not reflect accurately what happened [40].

Third, we had no data on other situational factors, such as the number of rounds fired by officers or which struck victims, each of which increase the odds of mortality [12], and therefore might render some of our observed statistical relationships spurious. Better measures, such as the location of the gunshot wound from California’s URSUS data, were able to provide a clearer view of the relationship between race and mortality. Here, we observed that 59.5% of Black victims were shot in the head or torso, versus 68.2% of non-Black victims. The inclusion of a covariate for wound location reduced the magnitude of the *Black victim* coefficient to practically nil (see S6 Table in S1 File). The FBI’s Law Enforcement Officers Killed and Assaulted (LEOKA) [41] program has long captured the distance between officer victims and their assailants, and it typically reveals that officers are more likely to be killed by firearms at shorter distances. Perhaps the observed differences in gunshot wound location among Black and non-Black victims in California were a partial function of the distance that separated them and officers at the time of the shootings. This is, of course, speculative–we lacked information on distance between officers and victims.

Fourth, our measure of access to trauma care was crude, as the state data on police shootings failed to include either the exact location of each incident or if/where the citizen received medical care. If such information was made available, future studies might endeavor to geolocate shootings and measure the distance in miles or average drive time to the nearest or the exact trauma center to provide for more specificity in spatial-temporal analyses [17, 18]. Finally, we lacked data on how quickly victims received medical aid. Future studies should attempt to determine and control for agencies’ policies (e.g., those regarding the rendering of first aid, transportation to hospitals, and use of tourniquets and hemostatic bandages).

## Conclusion

Researchers, policy makers, and law enforcement professionals desperately need more comprehensive data on police shootings. Rather than waiting on the federal government for such a system, which for decades criminologists have been clamoring for [42], more states should launch systems like those currently being used in Colorado, Texas, and California, each of which provide a more accurate count of deadly force incidents in their jurisdictions than older, federally operated systems ever did [13]. At the moment, California’s URSUS data is the gold standard–it tracks important information such as perceived versus actual weapons possessed by people, where on their bodies victims were shot, non-injurious shootings (i.e., misses), and incidents where people shoot at police officers [43]. Though it is understandable that researchers are leveraging the best available data (e.g., from *The Washington Post* or Fatal Encounters) to answer pressing policy and public health questions, we must recognize that fatalities are not a random sample of deadly force incidents and exercise more caution in drawing inferences from those data.

For instance, it has become common to rank agencies (or jurisdictions or other geographic units including entire states) in terms of their police-involved fatality rates using such data [2, 3, 16, 44–48]. This exercise is meant to identify agencies or places that are performing above and below average and to enable “public officials and the communities they represent to track the severity of the problem, devise preventive policies, and evaluate their efficacy” [3]. Yet, the ranked lists could look very different once nonfatal injurious police shootings are included. Similarly, researchers have turned to such data to assess whether some policy intervention or large-scale event reduced police-involved fatalities [49–52]. But here again, we might arrive at different conclusions if we focused on all instances of police use of deadly force rather than those that do in fact result in death.

Consider that when we compare jurisdictions in terms of reported crime rates, we must be mindful of the “dark figure”–the number of crimes that go unreported to police. If Community A has a lower crime rate than nearby Community B, is it because the crime rate was *actually* lower in Community A or was it because the people there reported crimes at a lower rate and/or agencies subsequently recorded them as less severe (i.e., misdemeanors instead of felonies)? Perhaps we should be thinking of nonfatal police shootings as the “dark figure” of police violence since we know so little about them relative to fatal shootings and other in-custody deaths. The good news is that it is possible to resolve this problem, and some states are already doing it (though as more states begin to adopt such systems, we must be mindful of the possibility for misclassifications/underreporting of non-firearm deaths) [53]. In the meantime, we must remember that an unknown dark figure is lurking behind much of the work being done on this topic. Researchers in this arena must start acknowledging this dark figure in all their future deadly force analyses.

## Supporting information

S1 File (DOCX)
