# Supplementary material for: Factors associated with police shooting mortality: A focus on race and a plea for more comprehensive data
Source: PLoS One. 2021 Nov 10;16(11):e0259024. doi: 10.1371/journal.pone.0259024 (PMC8580236; doi:10.1371/journal.pone.0259024)

**S1 File**

[Data Sources 2](#_Toc75524717)

[Table S1. Proportion of covariates that were fatal and nonfatal in each state. 7](#_Toc75524718)

[Table S2. Logistic regression models predicting the mortality of police shootings in the pooled sample (Ordinal Trauma care variable). 8](#_Toc75524719)

[Table S3. Logistic regression models predicting the mortality of police shootings in Florida. 9](#_Toc75524720)

[Table S4. Logistic regression models predicting the mortality of police shootings in Colorado. 10](#_Toc75524721)

[Table S5. Logistic regression models predicting the mortality of police shootings in Texas. 11](#_Toc75524722)

[Table S6. Logistic regression models predicting the mortality of police shootings in California. 12](#_Toc75524723)

[Figure S1. County-level mortality rates of police shootings in Florida. 13](#_Toc75524724)

[Figure S2. County-level mortality rates of police shootings in Colorado. 14](#_Toc75524725)

[Figure S3. County-level mortality rates of police shootings in Texas. 15](#_Toc75524726)

[Figure S4. County-level mortality rates of police shootings in California. 16](#_Toc75524727)

# **Data Sources**

## *Florida*

More information about and access to the publicly available *Tampa Bay Times*’ “Why Cops Shoot” database, including its sophisticated and diverse methodology, can be found at:

< <https://projects.tampabay.com/projects/2017/investigations/florida-police-shootings/>>.

## *Colorado*

The Colorado Officer-Involved Shootings data collection effort stems from Senate Bill 15-217, which was signed into law by Governor John Hickenlooper on May 5, 2015. The bill “requires any state or local law enforcement agency that employs a peace officer who is involved in an officer-involved shooting that results in a person suspected of criminal activity being shot at by the officer to report the information described below.”

“These agencies must report to the Division of Criminal Justice (DCJ) by September 1 of every year, any and all officer-involved shootings that occurred between July 1 and June 30 of the immediately preceding fiscal year. For example, please submit by September 1, 2020 officer-involved shootings that occurred between July 1, 2019 and June 30, 2020. Agencies must have also reported to DCJ any and all officer-involved shootings that occurred between January 1, 2010, and June 30, 2015.”

Given our research questions that sought to examine mortality (i.e., fatal versus injurious) among police shootings of people who were struck by gunfire, we excluded all “shoot and miss” cases.

More information, including the full-text of the Senate Bill 15-217, can be found at:

< <https://ors.colorado.gov/ors-coll-ois>>

Full reports examining officer-involved shooting incidents in Colorado that were prepared by the Colorado Division of Criminal Justice’s Office of Research and Statistics can be found at:

< <https://ors.colorado.gov/ors-reports>>

We emailed the Office of Research and Statistics to inquire about receiving a copy of the non-personally identifiable raw data. A representative provided us with data from January 1, 2010 through June 30, 2019.

*Texas*

The Texas data collection system was created by Article 2.139 of the Texas Code of Criminal Procedure. According to Article 2.139, “law enforcement agencies shall report all officer-involved injuries or deaths caused by the discharge of a firearm. For reports of this category (injuries or death to non-peace officer) you should complete a separate form for each officer who causes injury or death through the discharge of a firearm. Likewise, if more than one person is injured or killed by the discharge of a firearm by a peace officer, you should complete a separate form for each person injured or killed.”

Article 2.139 requires that agencies must submit a written or electronic ‘Peace Officer Involved Injuries or Death Form’ to the Texas Officer of the Attorney General (OAG) within 30 days of the incident in addition to posting a copy of the report on the agency’s website. The completed forms are made available to the Texas OAG, who is responsible for synthesizing its contents and creating annual reports.

The mandatory submission of all reports for officer-involved injuries/deaths caused by the discharge of a firearm began 1 September 2015. The publicly available shooting reports can be accessed at:

< <https://oagtx.force.com/oisreports/apex/OISReportsPage>>

## *California*

California’s URSUS police use of force reporting system was created by the state legislature, which passed Assembly Bill (AB) 71 and adding Government Code (GC) section 12525.2 in late 2015. It “mandates law enforcement agencies (LEA) in California to report use of force incidents that result in serious bodily injury or death or involved the discharge of a firearm. Effective January 1, 2016, all LEAs were required to begin collecting data on use of force incidents for submittal to the Department of Justice (DOJ).”

Similar to Colorado, we focused only on police officers’ discharges of a firearm and excluded all “shoot and miss” cases; only those shooting incidents where a person was shot and injured or killed were included. More information as well as the publicly available URSUS data can be accessed at:

< <https://openjustice.doj.ca.gov/data>>

## *Level I and II Adult Trauma Centers*

This variable, measured at the county level, was captured with the assistance of the Trauma Center Association of America (TCAA). TCAA’s website can be accessed at the following link:

< <https://www.traumacenters.org>>

TCAA’s website provides a search feature/map (“Map of US Trauma/Disaster Centers”) under the “Resources” tab. It can also be accessed at the following link:

< <https://www.traumacenters.org/page/TraumaCentersMap>>

We searched for and included only “Adult Level I” and “Adult Level II” trauma centers in each of the four states for our measure. The information in the search feature/map works in collaboration with the American College of Surgeons Committee on Trauma.

*County Urbanicity*

We obtained each county’s *urbanicity* from the US Department of Agriculture’s Economic Research Service. According to their website:

*ERS Rural-Urban Continuum Codes distinguish metropolitan (metro) counties by the population size of their metro area, and nonmetropolitan (nonmetro) counties by degree of urbanization and adjacency to metro areas. The Office of Management and Budget’s 2013 metro and nonmetro categories have been subdivided into three metro and six nonmetro groupings, resulting in a nine-part county classification. The codes provide researchers working with county data a more detailed residential classification, beyond a simple metro-nonmetro dichotomy, for the analysis of trends related to degree of rurality and metro proximity.*

Note that we reverse coded the Rural-Urban Continuum Codes so that higher scores reflected greater urbanicity. For more, see the following links:

< <https://www.ers.usda.gov/data-products/rural-urban-continuum-codes/documentation/>>

< <https://www.ers.usda.gov/topics/rural-economy-population/rural-classifications/what-is-rural/>>

Excel File data on 2013 RUCC from:

<<https://www.ers.usda.gov/data-products/rural-urban-continuum-codes.aspx>>

| Table S1. Proportion of covariates that were fatal and nonfatal in each state. | | | | | | | | | | | | | | | | |
| --- | --- | --- | --- | --- | --- | --- | --- | --- | --- | --- | --- | --- | --- | --- | --- | --- |
|  | Florida  *Tampa Bay Times*  *2009 – 2014* | | | | Colorado  *Office of Research and Statistics*  *2010 – 2019* | | | | Texas  *State Attorney General*  *2015 – 2019* | | | | California  *URSUS*  *2016 – 2019* | | | |
|  | Fatal | | Injurious | | Fatal | | Injurious | | Fatal | | Injurious | | Fatal | | Injurious | |
|  | N | [%] | N | [%] | N | [%] | N | [%] | N | [%] | N | [%] | N | [%] | N | [%] |
| **Overall** | 446 | [54] | 377 | [46] | 255 | [63] | 149 | [37] | 396 | [53] | 356 | [47] | 549 | [56] | 440 | [44] |
| *Race* |  |  |  |  |  |  |  |  |  |  |  |  |  |  |  |  |
| White | 201 | [61] | 129 | [39] | 140 | [65] | 75 | [35] | 159 | [57] | 120 | [43] | 176 | [57] | 131 | [43] |
| Black | 164 | [48] | 179 | [52] | 25 | [51] | 24 | [49] | 89 | [44] | 115 | [56] | 86 | [51] | 82 | [49] |
| Hispanic | 76 | [59] | 52 | [41] | 78 | [63] | 46 | [37] | 134 | [54] | 116 | [46] | 248 | [55] | 199 | [45] |
| Asian | 2 | [67] | 1 | [33] | 5 | [100] | 0 | [0] | 7 | [70] | 3 | [30] | 20 | [67] | 10 | [33] |
| Other | 0 | [0] | 1 | [100] | 3 | [100] | 0 | [0] | 5 | [83] | 1 | [17] | 19 | [51] | 18 | [49] |
| Undetermined | 3 | [17] | 15 | [83] | 4 | [50] | 4 | [50] | 2 | [67] | 1 | [33] | 0 | — | 0 | — |
| *Gender* |  |  |  |  |  |  |  |  |  |  |  |  |  |  |  |  |
| Male | 420 | [55] | 348 | [45] | 249 | [64] | 138 | [36] | 368 | [52] | 335 | [48] | 529 | [56] | 415 | [44] |
| Female | 26 | [48] | 28 | [52] | 6 | [35] | 11 | [65] | 28 | [57] | 21 | [43] | 20 | [44] | 25 | [56] |
| Undetermined | 0 | [0] | 1 | [100] | 0 | — | 0 | — | 0 | — | 0 | — | 0 | — | 0 | — |
| *Age* |  |  |  |  |  |  |  |  |  |  |  |  |  |  |  |  |
| 25 or younger | 105 | [41] | 152 | [59] | 66 | [58] | 47 | [42] | 92 | [44] | 116 | [56] | 125 | [46] | 146 | [54] |
| 26 – 35 | 131 | [55] | 106 | [45] | 70 | [59] | 48 | [41] | 124 | [53] | 111 | [47] | 185 | [57] | 142 | [43] |
| 36 – 45 | 80 | [60] | 53 | [40] | 66 | [70] | 28 | [30] | 85 | [58] | 61 | [42] | 126 | [58] | 92 | [42] |
| 46 or older | 127 | [69] | 57 | [31] | 52 | [68] | 25 | [32] | 93 | [59] | 64 | [41] | 113 | [65] | 60 | [35] |
| Undetermined | 3 | [25] | 9 | [75] | 1 | [50] | 1 | [50] | 2 | [40] | 3 | [60] | 0 | — | 0 | — |
| *Armed with* |  |  |  |  |  |  |  |  |  |  |  |  |  |  |  |  |
| Deadly weapon | 343 | [57] | 261 | [43] | 226 | [64] | 128 | [36] | 354 | [56] | 278 | [44] | 445 | [59] | 310 | [41] |
| Toy/BB gun | 25 | [60] | 17 | [40] | 0 | — | 0 | — | 0 | — | 0 | — | 34 | [63] | 20 | [37] |
| Unarmed | 67 | [43] | 89 | [57] | 12 | [43] | 16 | [57] | 42 | [35] | 78 | [65] | 70 | [39] | 110 | [61] |
| Undetermined | 11 | [52] | 10 | [48] | 17 | [77] | 5 | [23] | 0 | — | 0 | — | 0 | — | 0 | — |
| *Level I/II TC* |  |  |  |  |  |  |  |  |  |  |  |  |  |  |  |  |
| Yes | 375 | [52] | 347 | [48] | 206 | [63] | 123 | [37] | 260 | [51] | 250 | [49] | 474 | [56] | 369 | [44] |
| No | 71 | [70] | 30 | [30] | 49 | [65] | 26 | [35] | 136 | [56] | 105 | [44] | 75 | [51] | 71 | [49] |
| *Urbanicity* |  |  |  |  |  |  |  |  |  |  |  |  |  |  |  |  |
| Metro | 425 | [54] | 369 | [46] | 244 | [63] | 144 | [37] | 337 | [52] | 315 | [48] | 529 | [56] | 420 | [44] |
| Non-metro | 21 | [72] | 8 | [28] | 11 | [69] | 5 | [31] | 59 | [60] | 40 | [40] | 20 | [50] | 20 | [50] |
| *Mental Illness* |  |  |  |  |  |  |  |  |  |  |  |  |  |  |  |  |
| Yes | 128 | [71] | 53 | [29] | 22 | [73] | 8 | [27] | — | — | — | — | 29 | [54] | 25 | [46] |
| No | 310 | [50] | 316 | [50] | 221 | [62] | 135 | [38] | — | — | — | — | 520 | [56] | 415 | [44] |
| Undetermined | 8 | [50] | 8 | [50] | 12 | [67] | 6 | [33] | — | — | — | — | 0 | — | 0 | — |
| *Head/Torso shot* |  |  |  |  |  |  |  |  |  |  |  |  |  |  |  |  |
| Yes | — | — | — | — | — | — | — | — | — | — | — | — | 470 | [72] | 187 | [28] |
| No | — | — | — | — | — | — | — | — | — | — | — | — | 76 | [23] | 252 | [77] |
| Undetermined | — | — | — | — | — | — | — | — | — | — | — | — | 3 | [75] | 1 | [25] |

| Table S2. Logistic regression models predicting the mortality of police shootings in the pooled sample (Ordinal Trauma care variable and continuous urbanicity variable). | | | |
| --- | --- | --- | --- |
|  | *b* | SE | dy/dx  (95% CI) |
| Black victim ^a^ | -.294** | .105 | -.073  (-.124, -.022) |
| Hispanic victim ^a^ | .029 | .099 | .007  (-.040, .054) |
| Other victim ^a^ | .273 | .239 | .065  (-.044, .174) |
| Male victim | .068 | .159 | .017  (-.061, .094) |
| Age 26 – 35 ^b^ | .337*** | .086 | .084  (.042, .126) |
| Age 36 – 45 ^b^ | .536*** | .109 | .132  (.081, .184) |
| Age 46+ ^b^ | .661*** | .149 | .162  (.093, .231) |
| Weapon | .652*** | .105 | .162  (.111, .212) |
| Trauma care – 1 facility ^c^ | -.044 | .135 | -.011  (-.076, .055) |
| Trauma care – 2 facility ^c^ | -.071 | .140 | -.018  (-.086, .050) |
| Trauma care – 3+ facilities ^c^ | .106 | .129 | .026  (-.036, .088) |
| Urbanicity ^d^ | -.050 | .042 | -.012  (-.033, .008) |
| Colorado ^e^ | .126 | .168 | .030  (-.048, .109) |
| Texas ^e^ | -.229 | .128 | -.057  (-.119, .005) |
| California ^e^ | -.141 | .110 | -.035  (-.087, .018) |
| Intercept | -.169 | .353 | — |
| N | 2,892 | | |
| Wald χ^2^ | 121.05*** | | |
| ABBREVIATIONS: SE=Robust Standard Errors clustered on 246 counties; dy/dx = Average marginal effects showing the discrete change in the outcome (fatality) when moving from the reference category (estimated using *margins* command in Stata v15). Reference categories are ^a^ White victim, ^b^ Age 25 and under, ^c^ Zero trauma care facilities in the county, and ^e^ Florida, respectively. ^d^ *Urbanicity* is an ordinal variable that may take on values from 1 to 9 (mean: 8.38, SD: 1.25, range 1-9).  * *p* < .05, ** *p* < .01, *** *p* < .001 | | | |

| Table S3. Logistic regression models predicting the mortality of police shootings in Florida. | | | | | | | | |
| --- | --- | --- | --- | --- | --- | --- | --- | --- |
|  | | | | | | | | |
|  | | | | | | | | |
|  | Model 1 | | | | Model 2 | | | |
|  |  |  | |  |  |  | |  |
|  |  |  | |  |  |  | |  |
|  | b  (SE) | | dy/dx  (95% CI) | | b  (SE) | | dy/dx  (95% CI) | |
|  |  | |  | |  | |  | |
|  |  | |  | |  | |  | |
| Black victim ^a^ | -.531**  (.170) | | -.131  (-.212, -.050) | | .002  (.217) | | .001  (-.105, .106) | |
| Hispanic victim ^a^ | -.064  (.221) | | -.015  (-.119, .089) | | .303  (.272) | | .074  (-.054, .201) | |
| Other victim ^a^ | -.443  (1.066) | | -.109  (-.630, .412) | | -.213  (.934) | | -.053  (-.511, .404) | |
| Male victim | — | | — | | .240  (.302) | | .060 (-.088, .207) | |
| Age ^b^ | — | | — | | .024**  (.008) | | .006  (.002, .010) | |
| Mental illness ^c^ | — | | — | | .635***  (.186) | | .152  (.069, .234) | |
| Weapon | — | | — | | .283  (.187) | | .070  (-.021, .161) | |
| Trauma care | — | | — | | -.451  (.244) | | -.108  (-.219, .003) | |
| Urbanicity ^d^ | — | | — | | -.308  (.461) | | -.074  (-.286, .137) | |
| Intercept | .443***  (.116) | | — | | -.590  (.527) | | — | |
| N | 805 | | | | 772 | | | |
| Wald χ^2^ | 17.73*** | | | | 49.90*** | | | |
|  |  | | | |  | | | |
|  |  | | | |  | | | |
| ABBREVIATIONS: SE=Robust Standard Errors clustered on 53 (Model 1) and 52 counties (Model 2); dy/dx = Average marginal effects showing the discrete change in the outcome (fatality) when moving from the reference category (estimated using *margins* command in Stata v15). ^a^ Reference category is *White victim*. ^b^ *Age* is coded continuously (mean: 34.9, SD: 13.4, range 2 – 80). ^c^ *Mental illness* is coded dichotomously (1=yes; mean: .22, SD: .42). ^d^ *Urbanicity* is an ordinal variable that may take on values from 1 to 9 (mean: 8.55, SD: .87, range 4-9). * *p* < .05, ** *p* < .01, *** *p* < .001 | | | | | | | | |

| Table S4. Logistic regression models predicting the mortality of police shootings in Colorado. | | | | | | | | |
| --- | --- | --- | --- | --- | --- | --- | --- | --- |
|  | | | | | | | | |
|  | | | | | | | | |
|  | Model 1 | | | | Model 2 | | | |
|  |  |  | |  |  |  | |  |
|  |  |  | |  |  |  | |  |
|  | b  (SE) | | dy/dx  (95% CI) | | b  (SE) | | dy/dx  (95% CI) | |
|  |  | |  | |  | |  | |
|  |  | |  | |  | |  | |
| Black victim ^a^ | -.584*  (.296) | | -.141  (-.288, .005) | | -.714***  (.208) | | -.175  (-.276, -.074) | |
| Hispanic victim ^a^ | -.105  (.163) | | -.024  (-.099, .050) | | .004  (.188) | | .001  (-.084, .086) | |
| Other victim ^a^ | 15.405***  (.489) | | .349  (.276, .422) | | 14.321***  (.797) | | .361  (.280, .441) | |
| Male victim | — | | — | | .666  (.713) | | .158  (-.182, .498) | |
| Age ^b^ | — | | — | | .010  (.013) | | .002  (-.003, .008) | |
| Mental illness ^c^ | — | | — | | .504  (.426) | | .101  (-.058, .260) | |
| Weapon | — | | — | | .820  (.466) | | .195  (-.036, .427) | |
| Trauma care | — | | — | | -.177  (.234) | | -.038  (-.137, .062) | |
| Urbanicity ^d^ | — | | — | | .254  (.614) | | .058  (-.226, .342) | |
| Intercept | .624***  (.164) | | — | | -1.322  (.966) | | — | |
| N | 398 | | | | 359 | | | |
| Wald χ^2^ | 1020.57*** | | | | 1486.10*** | | | |
|  |  | | | |  | | | |
|  |  | | | |  | | | |
| ABBREVIATIONS: SE=Robust Standard Errors clustered on 26 counties; dy/dx = Average marginal effects showing the discrete change in the outcome (fatality) when moving from the reference category (estimated using *margins* command in Stata v15). ^a^ Reference category is *White victim*. ^b^ *Age* is coded continuously (mean: 34.6, SD: 12.1, range 16 – 73). ^c^ *Mental illness* is coded dichotomously (1=yes; mean: .08, SD: .27). ^d^ *Urbanicity* is an ordinal variable that may take on values from 1 to 9 (mean: 8.25, SD: 1.25, range 1-9).  * *p* < .05, ** *p* < .01, *** *p* < .001 | | | | | | | | |

| Table S5. Logistic regression models predicting the mortality of police shootings in Texas. | | | | | | | | |
| --- | --- | --- | --- | --- | --- | --- | --- | --- |
|  | | | | | | | | |
|  | | | | | | | | |
|  | Model 1 | | | | Model 2 | | | |
|  |  |  | |  |  |  | |  |
|  |  |  | |  |  |  | |  |
|  | b  (SE) | | dy/dx  (95% CI) | | b  (SE) | | dy/dx  (95% CI) | |
|  |  | |  | |  | |  | |
|  |  | |  | |  | |  | |
| Black victim ^a^ | -.538**  (.198) | | -.134  (-.229, -.038) | | -.369  (.190) | | -.092  (-.184, .000) | |
| Hispanic victim ^a^ | -.129  (.177) | | -.032  (-.118, .054) | | -.078  (.184) | | -.019  (-.109, .070) | |
| Other victim ^a^ | .817  (.500) | | .180  (-.009, .369) | | .840  (.522) | | .188  (-.014, .390) | |
| Male victim | — | | — | | -.434  (.243) | | -.106  (-.217, .006) | |
| Age ^b^ | — | | — | | .012  (.006) | | .003  (-.000, .006) | |
| Weapon | — | | — | | .844***  (.153) | | .207  (.137, .276) | |
| Trauma care | — | | — | | -.003  (.188) | | -.001  (-.092, .091) | |
| Urbanicity ^c^ | — | | — | | -.315  (.297) | | -.078  (-.218, .063) | |
| Intercept | .281*  (.120) | | — | | -.212  (.463) | | — | |
| N | 748 | | | | 743 | | | |
| Wald χ^2^ | 20.48*** | | | | 49.50** | | | |
|  |  | | | |  | | | |
|  |  | | | |  | | | |
| ABBREVIATIONS: SE=Robust Standard Errors clustered on 120 (Model 1) and 119 (Model 2) counties.  dy/dx = Average marginal effects showing the discrete change in the outcome (fatality) when moving from the reference category (estimated using *margins* command in Stata v15). ^a^ Reference category is *White victim*. ^b^ *Age* is coded continuously (mean: 34.7, SD: 12.8, range 4 – 84). ^c^ *Urbanicity* is an ordinal variable that may take on values from 1 to 9 (mean: 8.06, SD: 1.79, range 1-9). * *p* < .05, ** *p* < .01, *** *p* < .001 | | | | | | | | |

| Table S6. Logistic regression models predicting the mortality of police shootings in California. | | | | | | | | |
| --- | --- | --- | --- | --- | --- | --- | --- | --- |
|  | | | | | | | | |
|  | | | | | | | | |
|  | Model 1 | | | | Model 2 | | | |
|  |  |  | |  |  |  | |  |
|  |  |  | |  |  |  | |  |
|  | b  (SE) | | dy/dx  (95% CI) | | b  (SE) | | dy/dx  (95% CI) | |
|  |  | |  | |  | |  | |
|  |  | |  | |  | |  | |
| Black victim ^a^ | -.248  (.138) | | -.061  (-.128, .005) | | .016  (.162) | | .004  (-.074, .082) | |
| Hispanic victim ^a^ | -.075  (.140) | | -.018  (-.086, .049) | | .038  (.206) | | .009  (-.090, .108) | |
| Other victim ^a^ | .036  (.279) | | .009  (-.124, .142) | | -.064  (.326) | | -.016  (-.174, .142) | |
| Head/torso shot | — | | — | | 2.141***  (.171) | | .488  (.425, .551) | |
| Male victim | — | | — | | .195  (.296) | | .048  (-.096, .193) | |
| Age 26 – 35 ^b^ | — | | — | | .112  (.154) | | .028  (-.047, .103) | |
| Age 36 – 45 ^b^ | — | | — | | .251  (.183) | | .062  (-.026, .151) | |
| Age 46+ ^b^ | — | | — | | .658*  (.271) | | .157  (.037, .278) | |
| Mental illness | — | | — | | -.237  (.324) | | -.059  (-.217, .100) | |
| Weapon ^c^ | — | | — | | .823*  (.370) | | .202  (.032, .372) | |
| Trauma care | — | | — | | .225  (.211) | | .056  (-.047, .159) | |
| Urbanicity ^d^ | — | | — | | .223  (.414) | | .055  (-.147, .258) | |
| Intercept | .295**  (.099) | | — | | -2.617***  (.377) | | — | |
| N | 989 | | | | 943 | | | |
| Wald χ^2^ | 3.88 | | | | 358.02*** | | | |
|  |  | | | |  | | | |
|  |  | | | |  | | | |
| ABBREVIATIONS: SE=Robust Standard Errors clustered on 48 counties; dy/dx = Average marginal effects showing the discrete change in the outcome (fatality) when moving from the reference category (estimated using *margins* command in Stata v15). Reference categories are ^a^ White victim and ^b^ Age 25 and under, respectively. ^c^ *Weapon* was coded 1 if victim was armed or perceived by officers as armed. ^d^ *Urbanicity* is an ordinal variable that may take on values from 1 to 9 (mean: 8.54, SD: .95, range 2-9). There were 86 victims who officers perceived as armed but who were in fact unarmed. Results were substantively similar when these 86 victims were coded as 0 on *weapon*. *Mental illness* is coded dichotomously (1=yes; mean: .05, SD: .23).  * *p* < .05, ** *p* < .01, *** *p* < .001 | | | | | | | | |

# **Figure S1. County-level mortality rates of police shootings in Florida.**


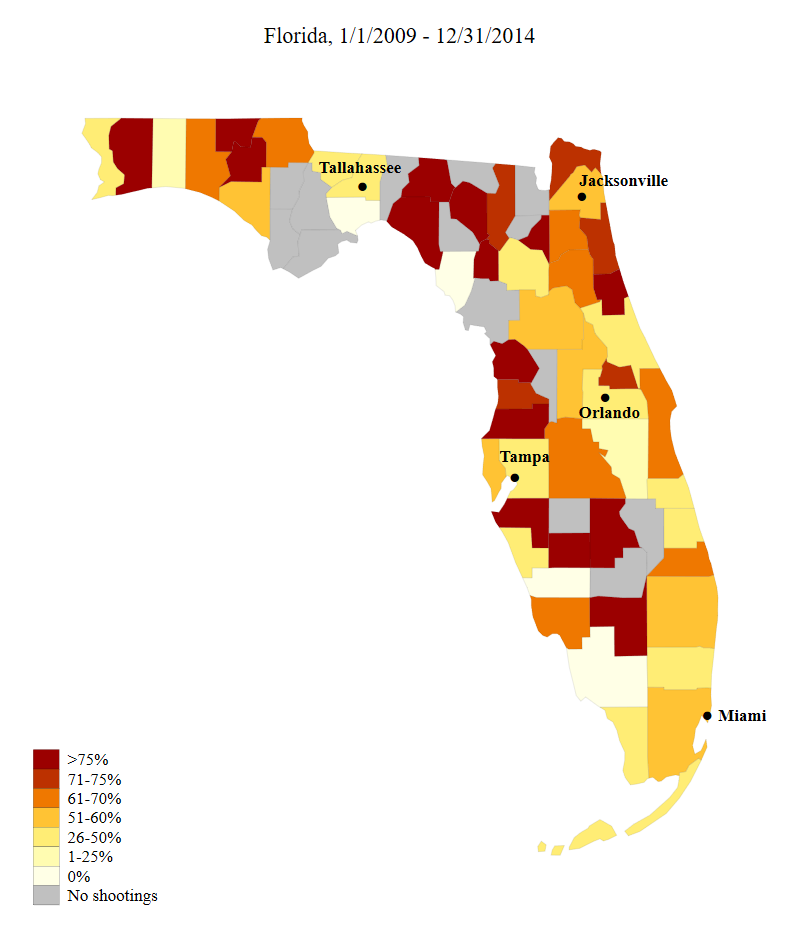


# **Figure S2. County-level mortality rates of police shootings in Colorado.**


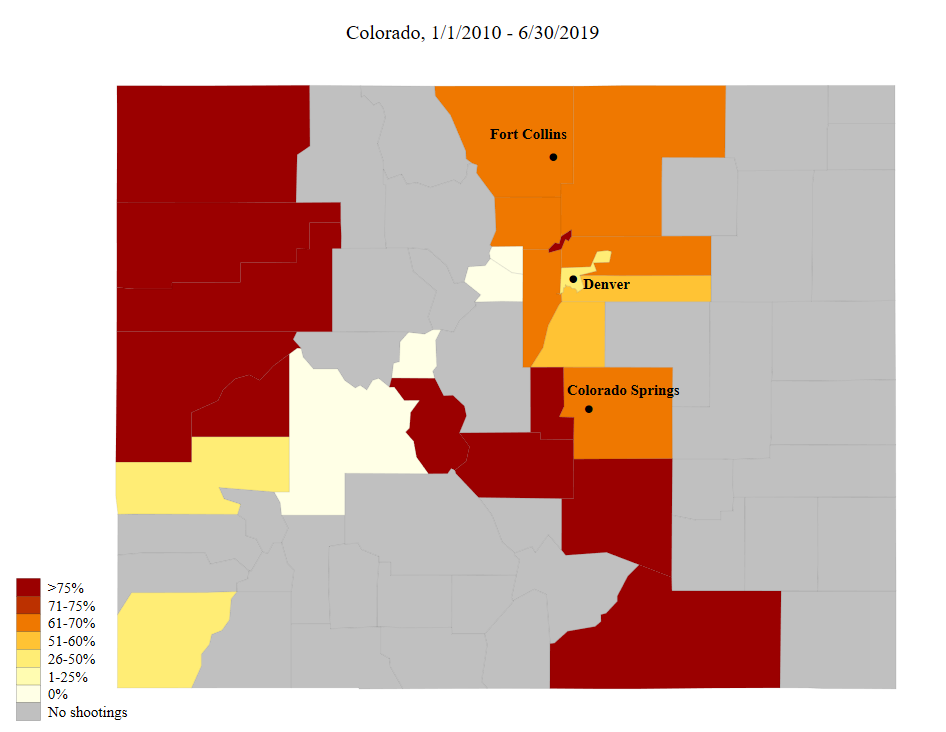


# **Figure S3. County-level mortality rates of police shootings in Texas.**


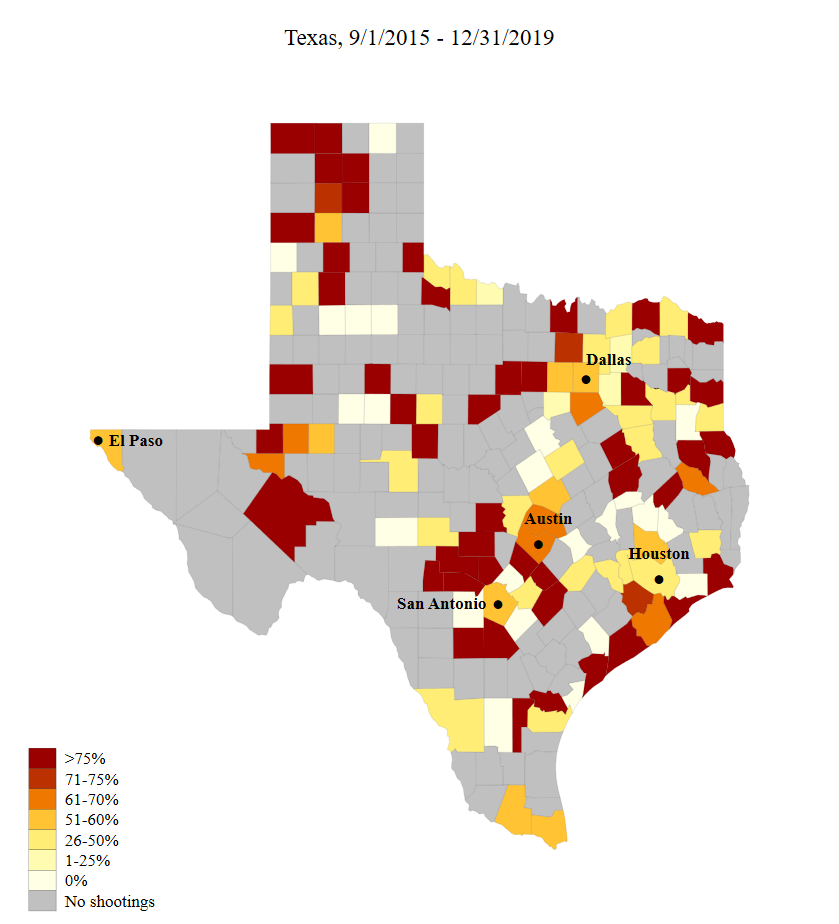


# **Figure S4. County-level mortality rates of police shootings in California.**


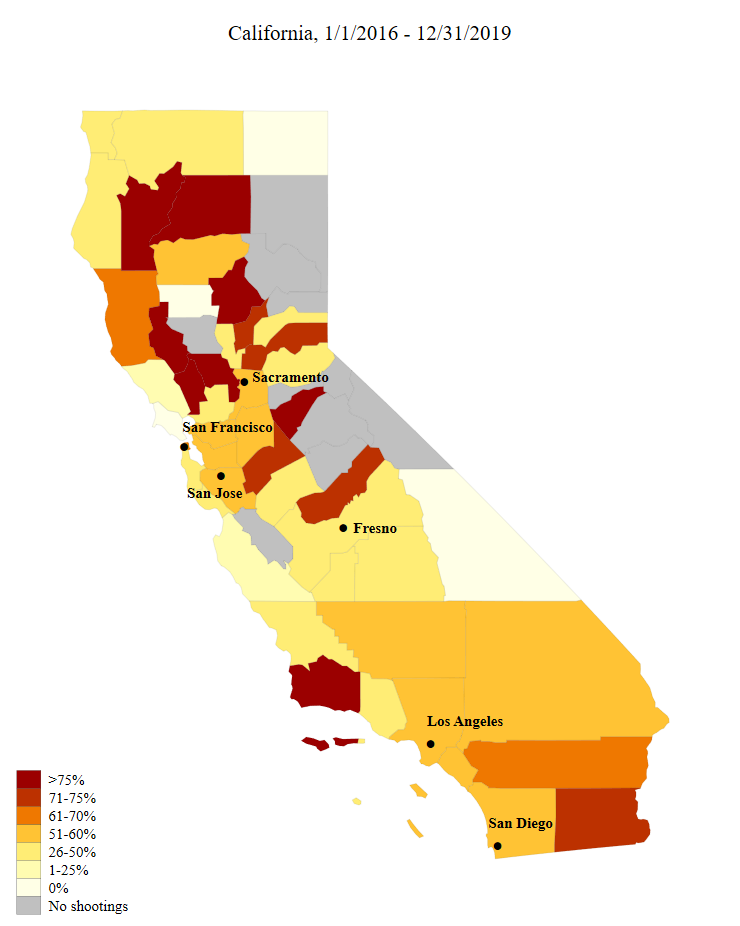

Supplement: S1 File — (DOCX) [file pone.0259024.s001.docx]
